# Why do preconception and pregnancy lifestyle interventions demonstrate limited success in preventing overweight and obesity in children? A scoping review protocol

**DOI:** 10.1371/journal.pone.0276491

**Published:** 2022-11-03

**Authors:** Kaat Philippe, Carla Perrotta, Aisling O’Donnell, Fionnuala M. McAuliffe, Catherine M. Phillips

**Affiliations:** 1 School of Public Health, Physiotherapy and Sports Science, University College Dublin, Belfield, Dublin, Ireland; 2 UCD Perinatal Research Centre, School of Medicine, University College Dublin, National Maternity Hospital, Dublin, Ireland; Edinburgh Napier University, UNITED KINGDOM

## Abstract

Adverse family-based lifestyle factors in the preconception period, pregnancy and early-childhood are major risk factors for childhood obesity and there is a growing consensus that early life interventions to prevent overweight and obesity in children are required. However, results from recent systematic reviews of preconception and pregnancy interventions have demonstrated mixed success. Therefore, this protocol presents a study aiming to summarise and evaluate complex preconception and pregnancy intervention components, process evaluation components, and authors’ statements, with a view to improving our understanding regarding their success and informing design or adaptation of more effective interventions to prevent childhood obesity. A scoping review will be conducted, using the frameworks of the JBI and Arksey and O’Malley. A two-step approach will be used to identify relevant literature: (1) systematic searches will be conducted in the databases PubMed, Embase and CENTRAL to identify all eligible preconception and pregnancy trials with offspring data; and (2) CLUSTER searches will be conducted to find linked publications to eligible trials (follow-ups, process evaluation publications). Two researchers will independently select studies, chart, and synthesise data. A qualitative thematic analysis will be performed in which statements related to process evaluation components and authors’ interpretations will be coded as “reasons”. A descriptive analysis will be performed to evaluate intervention complexity using a complex intervention framework (AHRQ series; Medical Research Council guidance). The results of this study, which will be discussed with an expert group as part of a consultation stage, aim to identify gaps and inform the design or adaptation of future preconception and pregnancy interventions and approaches to potentially increase success rates. We expect that our results, which will be submitted for publication in a peer-reviewed journal, will be of interest to researchers, families, and practitioners concerned with good preconception and prenatal care, and healthy child outcomes.

## Introduction

Childhood obesity is a major public health problem leading to short-term and long-term adverse health outcomes, reduced quality of life and high societal costs [1–5]. Adverse family-based lifestyle factors in the preconception period, pregnancy and early-childhood are major risk factors for childhood obesity [1], with consistent evidence highlighting the impact of maternal preconception BMI, gestational weight gain, and maternal tobacco use during pregnancy [6].

Interventions that involve human behaviour are typically complex [7]. Lifestyle, behavioural interventions aimed at having a transgenerational impact on child outcomes would require a complex intervention framework due to their necessary complexity concerning intervention, pathway, population, implementation and/or context [8]. Thus far, interventions during pregnancy targeting maternal lifestyle changes (nutrition, physical activity, etc) have demonstrated limited evidence for reducing offspring overweight and obesity outcomes in childhood (see reviews by Blake-Lamb et al., [9]; Louise et al., [10]; Raab et al., [11]). Recommendations for future interventions emphasize the need to start interventions before conception [9, 12]. So far, however, the evidence regarding effectiveness of interventions initiated during this period is very limited [12]. In addition, representation of fathers in family interventions during childhood is very low [13] and has yet to be explored in interventions before and during pregnancy.

To optimize future interventions, it is important to evaluate why current interventions during the perinatal period have demonstrated limited success in preventing overweight and obesity in childhood. Interventions, in general, may have limited effects either because of weaknesses in their design or because they are not properly implemented [14]. Moreover, Waters et al. [15] stated that “if reviews of intervention evidence are to be useful to decision-makers at all, contextual and implementation information is an essential, non-negotiable component of the review process”. Investigating process evaluation components (e.g., context, recruitment, reach, dose delivered, dose received, fidelity, implementation, participant’s attitudes toward the intervention) could thus provide information about limitations in current (pre)pregnancy interventions. Reported limitations in publications, as well as researchers’ interpretation regarding process evaluation components and their link with intervention outcomes can also be valuable sources worthwhile examining. In addition, interventions may also demonstrate limited success because they fail to tackle the multiple determinants of a complex condition. Complex intervention frameworks have been developed to aid development and evaluation of complex interventions [8, 16]. They could be a useful tool for evaluating current preconception and pregnancy interventions.

Taken together, a scoping review will be undertaken to identify complex intervention components, process evaluations components and authors’ statements that can help us understand the limited success of preconception and pregnancy lifestyle interventions on childhood overweight and obesity. The results of this study will help to inform more effective design or adaptation of future preconception and pregnancy interventions or approaches to prevent childhood overweight and obesity to improve their success rate.

## Materials and methods

A scoping review will be undertaken guided by the JBI guidance for scoping reviews [17] and by Arksey and O’Malley’s framework [18], which has been expanded and updated by Levac and colleagues [19], Daudt and colleagues [20], and Westphaln and colleagues [21]. This framework describes five key stages of conducting a scoping review and a sixth, optional stage: (i) specifying the research question; (ii) identifying relevant literature; (iii) selecting the studies; (iv) charting the data; (v) summarising, synthesizing, and reporting the results; and (vi) integrating expert consultation.

We chose to conduct a scoping review, because this type of systematic review identifies key concepts, research gaps, and evidence to inform practice, policymaking, and research [20]. Scoping reviews address questions beyond those related to intervention effectiveness and generate findings that can complement the findings of clinical trials [19]. Moreover, they use rigorous and transparent methods to identify and analyse relevant literature with the added advantage of including heterogeneous, methodologically diverse evidence [22]. The sixth stage of conducting a scoping review “integrating expert consultation” also allows to ensure that all decisions in the review process are appropriate and relevant, and to discuss and validate the obtained results.

This review protocol followed the Preferred Reporting Items for Systematic Review and Meta-Analysis Protocols (PRISMA-P) [23] guidelines that can be used for scoping reviews [24] (see “S1 File”). Any future deviations from the review protocol will be agreed by the reviewers and documented [17]. The publication resulting from this scoping review will follow the Preferred Reporting Items for Systematic Reviews and Meta-Analyses extension for scoping reviews checklist (PRISMA-ScR) guidelines [25]. The review team includes researchers with training and experience in childhood obesity, including diet and lifestyle (K.P., A.O.D., C.M.P. F.M.McA.), complex interventions in healthcare (F.M.McA., C.P.), and conducting reviews (A.O.D., C.P., C.M.P, K.P.).

### Stage 1: Specifying the research question

For the construction of the research questions, the PICO (population, interventions, comparators, outcomes) framework was applied. In this scoping review we are interested in:

- Population: individuals/families planning on becoming pregnant (i.e., of reproductive age, not currently pregnant, sexually active and expressed a conscious decision to conceive) [26] and pregnant mothers/expectant fathers and parents
- Interventions: lifestyle, behavioural interventions (i.e., nutrition, physical activity, counselling, health coaching)
- Comparators: a control group receiving no intervention or care as usual
- Outcomes: offspring anthropometric data at 1 month of age or older

Specifically, we are interested in why interventions with this population do not seem to show the desired results (preventing overweight and obesity in their offspring), as demonstrated in recent reviews (e.g., Raab et al., 2021). To understand this, we are interested in identifying/evaluating complex intervention components, process evaluations components and authors’ stated interpretations.

Taken together, the research questions in this scoping review are the following:

- What are the characteristics of the preconception and pregnancy interventions considering a complex intervention framework? (E.g., what is the evidence regarding the intervention complexity, pathway complexity, population complexity, implementation complexity and contextual complexity?)
- What can we learn from process evaluation components and authors’ statements for understanding why preconception and pregnancy interventions have demonstrated limited success in preventing overweight and obesity in children?

### Stage 2: Identifying relevant literature

A two-step approach will be used to identify relevant literature: first, all eligible preconception and pregnancy trials providing offspring data will be identified, then linked publications to eligible trials (follow-up publications, process evaluation publications) will be searched for.

We identified relevant pregnancy trials published between January 1990 to March 2020 from a review conducted by Raab et al. [11], who examined the association between lifestyle interventions during pregnancy and anthropometric outcomes during childhood. A total of 20 trials were identified. In short, they employed searches with keywords and Medical Subject Headings (or equivalent) across four concepts using the “AND” Boolean operator: Pregnancy, Lifestyle interventions, Offspring weight/weight-related parameters, and Randomized trials. Within each of the categories, key words were combined using the “OR” Boolean operator. The publications were limited to human studies and the English and German language. They also performed hand-searches of reference lists of included studies to avoid missing relevant publications. Examples of their searches can be consulted in their supplementary material.

Using the same methodology as Raab et al. [11], we will identify relevant pregnancy trials published after March 2020 (using the same search terms as Raab et al.) and all preconception trials published from January 1990 (pregnancy search terms replaced with preconception terms) through conducting systematic literature searches in the databases PubMed, Embase, and the Cochrane Central Register of Controlled Trials (CENTRAL). Following the JBI guidance for scoping reviews [17], we will not apply language restrictions a priori. Examples of our full electronic searches in the databases can be found in (“S2 File”). These will also be included in the supplementary material of our publication presenting the results of the review. The database searches will be performed in July 2022.

The search strategy does not contain relevant terms related to ‘process-evaluation’ or ‘interpretation” (such as “facilitators and barriers”, “implementation”, “process assessment”, “programme evaluation”), in the first place, because we anticipate that such terms are often not mentioned in the title and/or abstract, and possibly not even explicitly in the body of the text. Secondly, it is necessary to use the same methodology as Raab et al. [11] for ensuring consistency in the identification of relevant trials providing offspring anthropometric data.

In addition, CLUSTER (Citations, traced Lead authors, identified Unpublished materials, searched Google Scholar, tracked Theories, undertook ancestry searching for Early examples and followed up Related projects) searching [27] will be used to identify further eligible trials and linked publications to eligible trials (follow-up publications, process evaluation publications) [27]. When available, the name or acronym of the trial will be used as a keyword for finding these additional publications. To make sure no relevant process evaluation or follow-up publications are missed, the authors of eligible trials will be contacted. Experts in the field will also be consulted to identify other possible eligible trials (also see section “Stage 6: Integrating expert consultation”).

Search result citations will be imported into EndNote. Results will subsequently be uploaded to Covidence systematic review software [28] (available at www.covidence.org) for deletion of duplicates and screening of the publications. Records of the research protocol used for each database will be kept, and a PRISMA-ScR flow diagram will be produced to present an overview of the identification, screening, assessment of eligibility and selection processes.

### Stage 3: Selecting the studies

The study selection criteria will be pilot-tested by screening a random sample of 25 titles and abstracts in Covidence by two reviewers (K.P. and A.O.D.). Disagreements will be discussed immediately, possibly also with a third reviewer, until resolved by consensus. Subsequently, the remaining study titles and abstracts will be independently screened against eligibility criteria by two researchers (K.P. and A.O.D./TBD) to minimise potential bias in selecting studies. Any disagreements throughout the screening stage will be discussed, initially between the two reviewers and with a third author (C.M.P.) until resolved by consensus. Inclusion criteria may possibly also be refined along the way and upon agreement by the review team. If no abstract is available, the full paper will be retrieved. Following abstract review, the selected studies will proceed to full text screening by two reviewers (K.P. and TBD) for final selection. Reasons for exclusion will be recorded for full text screening, based on population, intervention, control, outcome, and study design (PICOS) characteristics (see “S3 File”).

Publications will be eligible if they meet the following criteria:

- Including individuals/families planning on becoming pregnant (i.e., of reproductive age, not currently pregnant, sexually active and expressed a conscious decision to conceive, [26]) and pregnant mothers/expectant fathers and parents (P)
- Describing lifestyle, behavioural interventions (i.e., nutrition, physical activity, counselling) (I), reporting also on a control group receiving no intervention or care as usual ((quasi-/cluster-) RCTs) (C)
- Evaluating outcomes including offspring anthropometric data at 1 month of age or older (O)

Qualitative and process-evaluation publications that are published alongside eligible trial publications are also eligible for inclusion in this review.

Publications will be excluded if the interventions are not conducted with humans, not initiated before or during pregnancy, not describing behavioural interventions (e.g., studies with only intake of nutritional supplements will be excluded) in a randomized design, and not providing offspring anthropometric data at 1 month of age or older. The cut-off of 1 month after delivery was chosen by Raab et al. to distinguish between infant/child outcomes and birth/neonatal outcomes. Protocols, reviews, methodological papers, opinion pieces, commentaries, books, book chapters and grey literature will be excluded; only publications from peer reviewed journals will be included as these enhance credibility of included studies and facilitate comparisons across the literature. An exception will be made for reports of process evaluations of eligible trials. These are not always published in peer-reviewed journals but offer a rich source of information about the implementation of the intervention and possible limitations. Information about process evaluations of eligible trials published in non-peer-reviewed journals will thus also be accepted for inclusion. If publications are in a language that none of the authors master and where a translator is not readily accessible or cannot be adequately translated using Google Translate [29], they will also be excluded.

### Stage 4: Charting the data

Data from the full text papers will be entered on to specially designed, pre-piloted and tailored data extraction sheets, to ensure a standardized way of summarising the details of the interventions [18]. The instruments will be developed involving input from all authors. The sheets will include data on trial and study characteristics, intervention complexity (guided by a complex intervention framework such as the AHRQ series on complex interventions systematic review [8]; Medical Research Council guidance [16]), as well as extracted process evaluation components, reported limitations, and author interpretations. A preliminary version of the data extraction sheets can be found in “S4 File”. For the trials that have been identified by Raab et al. before, the relevant information for this review can be found in their data extraction tables.

Following the recommendation of Peters et al. [17], two reviewers (K.P. and TBD) will extract at least two to three studies independently, discrepancies will be discussed, and any disagreements will be moderated by a third reviewer (C.M.P.). After this pilot test, one of the reviewers (K.P.) will complete the data extraction for the remaining studies. A portion of this data extraction will be checked for accuracy by a second reviewer. It may occur that as articles will be reviewed and understanding of the topic deepened, the data extraction form will be adapted throughout and that new types of data will be extracted. In this case, we will maintain a log of decision-making to document this process.

### Stage 5: Summarising, synthesizing, and reporting the results

A qualitative thematic analysis will be performed in which statements related to process-evaluation components, reported study limitations in publications and authors’ interpretations and hypotheses will be coded and labelled as “reasons”, following the example of Christie et al. [30] (they used the label “determinants”). The “reasons” will then be inductively grouped to form thematically similar categories and subcategories. This inductive approach was chosen because it will enable a comprehensive scoping of the information available in the publications, which will help us to gain valuable insights into the possible explanations why preconception and pregnancy interventions have demonstrated limited success in preventing overweight and obesity in children so far.

Two reviewers (K.P. and TBD) will first independently code and map a proportion of extracted “reasons” by hand, followed by a consensus meeting with each other and a third author (C.M.P.). This will be to ensure consensus regarding the proposed categories and subcategories. A coding sheet including examples will be developed at this point to facilitate further coding. When the reviewers will continue their independent coding, research team meetings will be held when needed to ensure consensus throughout the coding phase (i.e., researcher triangulation). Decision-making of each step of data analysis will be reported carefully.

A descriptive analysis will be performed to evaluate the complexity of the included interventions.

In line with the PRISMA-ScR guidelines [25], the study findings will be summarized, reported, and contextualised within the research questions and aims, and interpreted within the context of knowledge gaps, future research needs/directions/recommendations, practice, and policy. They will be presented through tables, charts, and narrative summaries. Following scoping review methodology, an evaluation of study quality will not be performed and thus not presented [17].

### Stage 6: Integrating expert consultation

The sixth stage of the Arksey and O’Malley framework [18] “integrating expert consultation” was suggested by the authors as an optional stage, but Levac et al. [19] argue that it should be considered a required component because it adds methodological rigor. A first element of this stage involves consulting with key stakeholders in order to identify any further references and studies that they feel should be included. Furthermore, it allows opportunity to gather experts’ feedback regarding the scoping review findings. This additional consultation with content experts ensures that the search strategy includes all the appropriate terms and enhances the relevance of the research overall.

For this scoping review, we purposefully put together a review team with expertise in the field and obtained additional advice from the University librarian with expertise in conducting reviews. Moreover, this scoping review was conducted within the European project “EndObesity” which aims to develop, implement, and evaluate innovative, multi-disciplinary strategies for prevention of childhood obesity by targeting family-based lifestyle factors in the pre-conception period, pregnancy, and early childhood, covering the first 1000 days of life. The project brings together different stakeholders from various countries, such as researchers, municipal/governmental health care services, educational stakeholders, industry partners and parent childhood organisations with expertise in different fields (psychology, gynaecology, paediatrics, nutritional sciences, epidemiology, interventions, and implementation) who represent an ideal expert group for consultation.

The expert group will be consulted at three occasions. The first consultation took place at the annual meeting of the EndObesity consortium in June 2022, where the first official version of the scoping review protocol was presented. The experts from this consortium were invited to provide feedback on the protocol and suggestions for improvement. This ensured that the review aims and methods were relevant and adequate. Only minor adjustments related to the selection of studies (i.e., the search strategy for process evaluation publications) were made to the protocol based on their feedback. The second consultation is planned at the end of stage 3 “selecting the studies”. A list of included eligible intervention trials will be shared with the group and they will be asked to evaluate the completeness of the list. If the experts identify trials that should be eligible and have not been identified with the systematic and CLUSTER searches, they will be included in the review. If applicable, the number of added trials will also be reported in the flow diagram of the study selection. The third consultation is planned when the preliminary results are available. At this point, we will conduct a focus group where the results of the review will be presented to the consultation group and they will be asked to discuss the results (their opinions about how we mapped and conceptualized the results as well as the content of the results, whether/how likely they will implement each component/consider in planning future interventions or research, etc.). A list of pre-specified questions to guide discussion and receive specific feedback will be prepared to facilitate the focus group. The focus group will be recorded and transcribed at verbatim, whereafter transcriptions will be analysed and coded. Field notes will be taken throughout the focus group to supplement the thematic analysis. Stakeholder evidence will be presented in the review publication. If difficulty is encountered in organising a focus group due to practical reasons, we will consider sending a survey with similar content (open questions) to the different experts so they can complete it at a time that suits them. Survey responses would be analysed thematically.

In addition, we are also considering consulting other stakeholders like the researchers of the identified (pre)pregnancy trials, expectant or young parents (mothers and fathers), and health care professionals to obtain their input on our preliminary results and their view on the matter. Focus groups, interviews or online surveys may be appropriate for this. If time and resources allow this additional consultation, the results will also be presented in the review publication.

All stages of this scoping review are planned to be completed by the end of 2022.

## Discussion

So far, preconception and pregnancy interventions have demonstrated limited and mixed success in preventing childhood overweight and obesity in children [9, 11, 12]. This scoping review therefore aims to identify and evaluate complex intervention components, process evaluations components and authors’ statements that can help us understand why preconception and pregnancy interventions have demonstrated limited success. The originality and key feature of this scoping review lies in the fact that it goes beyond merely evaluating the effectiveness of interventions, as has been done in previous reviews. Instead, various elements of the intervention studies will be evaluated and mapped together in order to get insight regarding limitations in the current studies, but also elements with potential. To our knowledge, this will also be one of the first reviews that will combine insights from preconception and pregnancy interventions.

The results of this study, which will be discussed with experts as part of a consultation stage, aim to identify gaps and inform the design or adaptation of future preconception and pregnancy interventions and approaches to potentially increase success rates. Alternative strategies to prevent the development of overweight and obesity in children may also be suggested if appropriate or applicable based on the results. We expect that our results will be of interest not only to researchers, but also families, individuals, and practitioners concerned with good preconception and prenatal care, and prevention of childhood overweight and obesity.

## Supporting information

S1 File PRISMA-P checklist. (PDF)

S2 File Search strategy examples. (PDF)

S3 File Study eligibility checklist. (PDF)

S4 File Preliminary data extraction sheets. (PDF)
