# Supplementary material for: Why do preconception and pregnancy lifestyle interventions demonstrate limited success in preventing overweight and obesity in children? A scoping review protocol
Source: PLoS One. 2022 Nov 3;17(11):e0276491. doi: 10.1371/journal.pone.0276491 (PMC9632912; doi:10.1371/journal.pone.0276491)
Supplement: S2 File — (PDF) [file pone.0276491.s002.pdf]

Supplementary file 2. Search strategy examples

Pregnancy trials

Concepts	Terms PubMed	Terms Embase
Population:<br>Pregnant mothers/ expectant fathers and parents	(((((((((("pregnancy") OR "pregnant") OR "prenatal") OR "antenatal") OR "gestation") OR "gestational"))	(pregnan* or "prenatal" or "antenatal" or gestation*)
Interventions:<br>Behavioural, lifestyle interventions	AND<br>((("intervention") OR "trial") OR "study"))<br>AND<br>(((((((((((((((((("exercise") OR "exercising") OR "physical activity") OR "diet") OR "dieting") OR "dietary") OR "nutrition") OR "nutritional") OR "lifestyle") OR "behavior") OR "behavioral") OR "behaviour") OR "behavioural") OR "counsel") OR "counseling") OR "counselling") OR "monitor") OR "monitoring") OR "advice") OR "advise"))	and<br>(interven* or "trial" or "study")<br>and<br>(exercis* or "physical activity" or diet* or nutrition* or "lifestyle" or behavio* or counsel* or monitor* or "advice" or "advise")
Outcomes:<br>Child anthropometric data	AND<br>((((((((("infant") OR "infancy") OR "child") OR "children") OR "childhood") OR "offspring") OR "adolescence") OR "adolescent") OR "toddler") OR "youth"))<br>AND<br>(((((((((((((((((((("weight") OR "bmi") OR "body mass index") OR "percent") OR "percentile") OR "z score") OR "overweight") OR "obesity") OR "obese") OR "adiposity") OR "adipose") OR "anthropometry") OR "anthropometric") OR "metabolism") OR "metabolic") OR "circumference") OR "body fat") OR "fat mass") OR "lean mass") OR "body composition") OR "skinfold thickness"))	and<br>(infan* or child* or "offspring" or adolescen* or "toddler" or youth")<br>and<br>("weight" or "bmi" or "body mass index" or percent* or "z score" or "overweight" or obes* or adipos* or anthropometr* or metabol* or "circumference" or "body fat" or "fat mass" or "lean mass" or "body composition" or "skinfold thickness")
Comparators:<br>Control group: quasi-/cluster-RCT's	AND<br>((((("random") OR "randomly") OR "randomized") OR "randomised") OR "rct")	and<br>(random* or "rct")
	**Filters used**: "Humans", "Publication data: from 2020/3/1-now", "Clinical Trial", and "Randomized Controlled Trial"	**Filters used:** "Human", "Publication years: 2020, 2021, 2022, "Controlled study", and "Randomized Controlled Trial"

Note. Search strategy based on the one of Raab et al. (2021).

Preconception trials

Concepts	Terms PubMed	Terms Embase
Population:<br>Individuals/families planning on becoming pregnant	((((((((((((((((((((((((((care, preconception[MeSH Terms])) OR (preconception care[MeSH Terms])) OR (conception[MeSH Terms])) OR (Prepregnan*)) OR (pregravid*)) OR (pre-pregnan*)) OR (pre-gravid*)) OR (preconception)) OR (pre-conception)) OR (periconception)) OR (peri-conception)) OR (interconception)) OR (inter-conception)) OR (interpregnan*)) OR (inter-pregnan*)) OR (internatal)) OR (perinatal)) OR (peri-natal)) OR (family planning[MeSH Terms]))	(prepregnan* or pregravid* or pre-pregnan* or pre-gravid* or “preconception” or “pre-conception” or “periconception” or “peri-conception” or “interconception” or “inter-conception” or interpregnan* or inter-pregnan* or “internatal” or “perinatal” or “peri-natal” or “family planning”)
Interventions:<br>Behavioural, lifestyle interventions	AND<br>((("intervention") OR "trial") OR "study"))<br>AND<br>(((((((((((((((((("exercise") OR "exercising") OR "physical activity") OR "diet") OR "dieting") OR "dietary") OR "nutrition") OR "nutritional") OR "lifestyle") OR "behavior") OR "behavioral") OR "behaviour") OR "behavioural") OR "counsel") OR "counseling") OR "counselling") OR "monitor") OR "monitoring") OR "advice") OR "advise"))	and<br>(interven* or "trial" or "study")<br>and<br>(exercis* or "physical activity" or diet* or nutrition* or "lifestyle" or behavio* or counsel* or monitor* or "advice" or "advise")
Outcomes:<br>Child anthropometric data	AND<br>((((((((("infant") OR "infancy") OR "child") OR "children") OR "childhood") OR "offspring") OR "adolescence") OR "adolescent") OR "toddler") OR "youth"))<br>AND<br>((((((((((((((((((("weight") OR "bmi") OR "body mass index") OR "percent") OR "percentile") OR "z score") OR "overweight") OR "obesity") OR "obese") OR "adiposity") OR "adipose") OR "anthropometry") OR "anthropometric") OR "metabolism") OR "metabolic") OR "circumference") OR "body fat") OR "fat mass") OR "lean mass") OR "body composition") OR "skinfold thickness"))	and<br>(infan* or child* or "offspring" or adolescen* or "toddler" or youth")<br>and<br>("weight" or "bmi" or "body mass index" or percent* or "z score" or "overweight" or obes* or adipos* or anthropometr* or metabol* or "circumference" or "body fat" or "fat mass" or "lean mass" or "body composition" or "skinfold thickness")
Comparators:<br>Control group: quasi-/cluster-RCT’s	AND<br>((((("random") OR "randomly") OR "randomized") OR "randomised") OR "rct")	and<br>(random* or "rct")
	**Filters used**: “Humans”, “Clinical Trial”, and “Randomized Controlled Trial”	**Filters used:** “Human”, “Controlled study”, and “Randomized Controlled Trial”

Note. Search strategy based on the one of Raab et al. (2021).
