# Supplementary material for: Why do preconception and pregnancy lifestyle interventions demonstrate limited success in preventing overweight and obesity in children? A scoping review protocol
Source: PLoS One. 2022 Nov 3;17(11):e0276491. doi: 10.1371/journal.pone.0276491 (PMC9632912; doi:10.1371/journal.pone.0276491)
Supplement: S3 File — (PDF) [file pone.0276491.s003.pdf]

Supplementary file 3. Study eligibility checklist

**Inclusion Criteria**	**Yes/No**
Population: Individuals/families planning on becoming pregnant or pregnant mothers/expectant fathers and parents at time of the start of the intervention?	
Interventions: Behavioural, lifestyle intervention?	
Comparators: Control group (no intervention or care as usual): quasi-/cluster-RCT?	
Outcomes: Child anthropometric data after one month of age reported?	
Study: Human research?	
Study: Peer-reviewed journal publication? (Exception for process evaluation reports)?	

Note. If the answer is “No” to one or more of the above questions, the publication is not eligible for inclusion in the review.
