# Supplementary material for: Why do preconception and pregnancy lifestyle interventions demonstrate limited success in preventing overweight and obesity in children? A scoping review protocol
Source: PLoS One. 2022 Nov 3;17(11):e0276491. doi: 10.1371/journal.pone.0276491 (PMC9632912; doi:10.1371/journal.pone.0276491)
Supplement: S4 File — (PDF) [file pone.0276491.s004.pdf]

Supplementary file 4. Preliminary data extraction sheets

Sheet 1. Trial and study characteristics

**Source**	Trial ID (Number created by review author)
	Trial name
	Study ID (First author, Publication year)
	Citation
**Study Methods**	Study design **(**RCT/RCT cluster/...)
	Randomization
	Allocation sequence concealment
	Blinding
	Enrolment start and end dates
**Rationale/Theoretical foundation for intervention**	
**Aim(s) intervention**	Primary aim(s), Secondary aim(s)
**Participants** (intervention and control group(s))	Country/ies and/or region(s) from which participants were recruited
	Setting(s) **(**Hospital/Home/Community/…)
	Number of subjects (mothers/fathers/...) eligible for inclusion
	Study eligibility criteria
	Exclusion criteria for participation
	Number of subjects randomized
	Number of subjects who completed the intervention
	Attrition (subject dropout) at the end of intervention
	BMI class at start intervention (% of n Underweight/Normal/OW/OB)
	Ethnicity
	Socio-economic status
	Other characteristics or comorbidities (e.g., (at risk for) gestational diabetes mellitus, at risk for high GWG, smoking, lifestyle at start intervention…)
	Number of subjects at follow-up
	Timing follow-up subject
	Attrition subjects at follow-up
	Number of children at follow-up
	Timing follow-up children (age in months)
	Attrition children at follow-up

**Intervention content**	Number of intervention groups
	Timing intervention (Prepregnancy and/or Pregnancy and/or Postpartum)
	Specific start intervention (e.g., gestational week)
	Duration
	Frequency
	Nature of intervention – behaviours targeted
	Techniques and materials used
	Supporting theory/ies for intervention components and techniques used
	Delivery (online/face-to-face/phone/SMS/..; individual/group; ...)
	Intervention deliverers
	To whom the intervention was directed
	Whose behaviour/action the intervention intended to change
	Control intervention content (no intervention/care as usual/control intervention… + description)
**Outcomes**	Outcomes measured in subjects + measurement tool + timing
	Significant results in subjects (means + SDs of intervention vs. control group, *p*-value)
	Outcomes measured in children + measurement tool + timing
	Significant results in children (means + SDs of intervention vs. control group, *p*-value)
**Miscellaneous**	Comments review authors
	Correspondence required (Yes/No + reason)
	References to other relevant studies

Note: This sheet may still be split in separate sheets if this would facilitate the data extraction process.

Sheet 2. Intervention complexity assessed with the intervention Complexity Assessment Tool for Systematic Reviews (iCAT_SR; Lewin et al., 2017)

**Source**	Trial ID (Number created by review author)
	Trial name
1. **Active components** included in the intervention, in relation to the comparison	1.a Judgement: More than one component and delivered as a bundle/ More than one component/ One component/ Varies
	1.b Support for judgement (insert text from publication and/or related assumptions)
2. **Behaviour or actions** of intervention recipients or participants to which the intervention is directed	2.a Judgement: Multi-target/ Dual target/ Single target/ Varies
	2.b Support for judgement
3. **Organisational levels and categories** targeted by the intervention	3.a Judgement: Multi-level/ Multi-category/ Single category
	3.b Support for judgement
4. The **degree of tailoring intended or flexibility** permitted across sites or individuals in applying or implementing the intervention	4.a Judgement: Highly tailored – flexible/ Moderately tailored – flexible/ Inflexible/ Varies
	4.b Support for judgement
5. The **level of skill** required by those **delivering** the intervention in order to meet the intervention objectives	5.a Judgement: High level skills/ Intermediate level skills/ Basic skills/ Varies
	5.b Support for judgement
6. The **level of skill required** for the targeted behaviour when entering the included studies by those **receiving** the intervention, in order to meet the intervention objectives	6.a Judgement: High level skills/ Intermediate level skills/ Basic skills/ Varies
	6.b Support for judgement
7. The **degree of interaction between intervention components**, including the independence/interdependence of intervention components	7.a Judgement: High level interaction/ Moderate interaction/ Independent/ Varies/ Unclear or unable to assess
	7.b Support for judgement
8. The degree to which the effects of the intervention are **dependent on the context or setting in which it is implemented**	8.a Judgement: Highly context dependent/ Moderately context dependent/ Independent of context/ Varies/ Unclear or unable to assess
	8.b Support for judgement
9. The **degree to which the effects of the intervention are changed by recipient or provider factors**	9.a Judgement: Highly dependent on individual-level factors/ Moderately dependent on individual-level factors/ Largely independent of individual-level factors/ Varies/ Unclear or unable to assess
	9.b Support for judgement
10. The **nature of the causal pathway** between the intervention and the outcome it is intended to effect	10.a Judgement: Pathway variable, long/ Pathway linear, long/ Pathway linear, short/ Varies /Unclear or unable to assess
	10.b Support for judgement
**Miscellaneous**	Comments review authors

Dimensions and assessment levels iCAT_SR retrieved from: Lewin S, Hendry M, Chandler J, Oxman AD, Michie S, Shepperd S, Reeves BC, Tugwell P, Hannes K, Rehfuess EA, Welch V. Assessing the complexity of interventions within systematic reviews: development, content and use of a new tool (iCAT_SR). BMC medical research methodology. 2017 Dec;17(1):1-3.

Sheet 3. Data related to process evaluations, study limitations, and author interpretations

<table>
<tr><td rowspan="5">Source</td><td>Trial ID (Number created by review author)</td></tr>
<tr><td>Trial name</td></tr>
<tr><td>Study ID (First author, Publication year)</td></tr>
<tr><td>Citation</td></tr>
<tr><td>Separate process evaluation (PE)/qualitative paper or mixed-method paper? (Yes/No)</td></tr>
<tr><td rowspan="9">If "Yes" on the previous question, complete the following:</td><td>Aim(s) of the PE/qualitative evaluation</td></tr>
<tr><td>Timing of the evaluation in relation to the intervention (Before/ During/ After)</td></tr>
<tr><td>Content of the evaluation (e.g., acceptability, …)</td></tr>
<tr><td>Framework or theory used</td></tr>
<tr><td>Method(s) (Interviews/ Focus groups/ Survey/ Observations/ …)</td></tr>
<tr><td>Evaluation deliverers (who are the interviewers? - (in)dependency to intervention)</td></tr>
<tr><td>Description subjects</td></tr>
<tr><td>Number of subjects</td></tr>
<tr><td>Extra comments of review authors</td></tr>
<tr><td rowspan="3">Findings*</td><td>Components related to the Rationale/Theory or Study design*
Examples:
- Sample size
- Dose
- Timing and/or duration of intervention
- Behaviors targeted
- Techniques used
- Population targeted and selected – biases?
- Effects on maternal outcomes
- Measures used
- Other</td></tr>
<tr><td>Components related to the process (process evaluation components)*
Examples:
- Context
- Recruitment
- Reach
- Dose delivered
- Dose received/Adherence
- Participants' attitudes/acceptability
- Experiences of participants, intervention deliverers and researchers
- Feasibility
- Retention
- Fidelity
- Drop-out
- Attrition
- Other</td></tr>
<tr><td>Other components*</td></tr>
</table>

*Note: These data will most likely be extracted and coded directly in the qualitative research tool Nvivo – not in a separate Excel data extraction sheet. The extracted data will be inductively grouped to form thematically similar categories and subcategories. The elements in the sheet above are only illustrative of possible categories and subcategories that will be generated.
